# Focal neuropathologies in the brain of COVID-19-infected humans: inflammation, primary gliovascular failure and microglial dysfunction

**DOI:** 10.1038/s41392-025-02351-3

**Published:** 2025-08-25

**Authors:** Peter Illes, Hai-Yan Yin, Yong Tang

**Affiliations:** 1 Rudolf Boehm Institute for Pharmacology and Toxicology, University of Leipzig, Leipzig, Germany; 2 International Joint Research Centre on Purinergic Signalling, School of Acupuncture and Tuina, Chengdu University of Traditional Chinese Medicine, Chengdu, China; 3 School of Health and Rehabilitation, Chengdu University of Traditional Chinese Medicine, Chengdu, China

**Keywords:** Cellular neuroscience, Neurological disorders

Dénes and his co-workers recently published a paper in *Nature Neuroscience*, documenting that neurological abnormalities in COVID are based on microglial dysfunction in the brain.^1^ In case of acute respiratory syndrome of COVID infection, the central nervous system symptomatology significantly contribute to the severity of this disease. The authors used an autopsy platform allowing morphological and biochemical/molecular biological investigations in postmortem mirror blocks prepared from the brain and peripheral organs of 13 COVID and 23 non- COVID-infected patients.

Microglia are resident macrophages of the brain, representing a first defense wall in the case of any type of bacterial or viral infection.^2^ They possess G protein-coupled purinergic P2Y12 receptors (Rs) that sense ADP, arising from the enzymatic degradation of ATP that flows out from glial/neuronal cells after damage, caused by infections or metabolic disturbances. P2Y12Rs are localized at the membrane of the cytoplasmic processes of microglia; increased levels of locally generated ADP attract the active elongation of these processes to approach the sites of injury and later the migration of ameboid microglia to these sites.^3^

Immunofluorescence was used for quantification of the levels of P2Y12Rs in the gyrus rectus of the frontal cortex and the medulla oblongata; a lower expression of this receptor was reported in COVID-afflicted microglia. The resulting shortfall in the recognition of ADP interfered with the defensive function of microglial cells. In the brain parenchyma, the P2Y12R is specific for microglia and is not found on astrocyte processes. Down-regulation of P2Y12R expression in microglia correlated with focal lesions of COVID brains. Disintegration of vascular endothelium was observed at sites of microglial P2Y12R loss. Another important purinergic receptor in microglia is the P2X7R, which was not assessed in this study, but is included in Fig. 1.

**Fig. 1:**
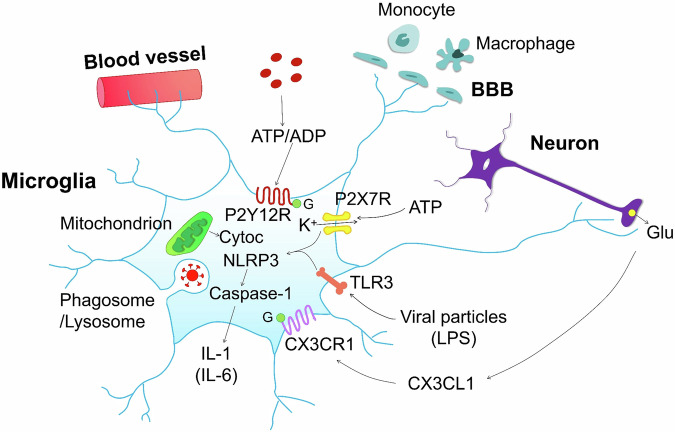
Microglia is a central player in the pathological events developing in the brain of COVID-19-infected patients. The infection-induced injury takes place preferentially in the medulla oblongata with its cardiovascular and respiratory centers whose disturbance may lead to severe danger for life. Microglial cells project their cytoplasmic processes to blood vessels and cause disintegration of vascular endothelium. They also project to the blood-brain-barrier (BBB), and by causing its breakdown, allow the entry of monocytes/macrophages into the cerebrospinal fluid (CSF). Eventually, microglia send their projections to glutamatergic neurons and engulf synaptic terminals/axons initiating the disintegration of the myelin sheath. Microglia are endowed with a number of receptors. The G protein (G)-coupled typical 7 transmembrane region P2Y12 receptor (R) senses ADP, which is generated by enzymatic degradation of extracellular ATP, flowing out from neuronal and non- neuronal cells through their damaged cell membrane. The P2Y12R induces the elongation of microglial processes to the sites of injury and later the migration of activated, ameboid microglia to these sites. COVID infection decreases the density of P2Y12Rs interfering with the recognition of ADP and thereby with the defensive microglial functions. The ligand-gated P2X7R is a cationic channel (permeable to Na^+^, K^+^ and Ca^2+^) which binds millimolar concentration of ATP; the loss of intracellular K^+^ is the first step in activating the NLRP3 inflammasome. In co- operation with the toll-like receptor 3 (TLR3) responding to e.g., the bacterial endotoxin lipopolysaccharide (LPS), or in the case of COVID-disease, virus particles, the activation of the proteolytic enzyme caspase-1 ensues. This results in the production and release of the pro-inflammatory cytokine interleukin-1 (IL-1). IL-6 also joins IL-1 to promote neuroinflammation. The G protein-coupled 7 transmembrane region chemokine receptor CX3CR1 (fractalkine-R) is stimulated by CX3CL1, released from neurons. The microglial mitochondria contain lowered levels of cytochrom c (Cytc) as a consequence of its outflow into the cytoplasm as a marker of apoptosis. Microglia phagocytose viruses; phagolysosomes arise by the fusion of phagosomes and lysosomes containing the virus, and myelin basic protein from the disintegrated myelin sheath of neuronal axons

Quantification of IgG extravasation was used as a marker of blood-brain-barrier (BBB) breakdown during COVID-disease; this damage was more severe in the medulla oblongata than in any other part of the brain. At these sites interleukin-1 (IL-1) and -6 were potent drivers of inflammation and recruited neutrophils, monocytes and macrophages passing the damaged BBB on their way from the peripheral circulation.^4^ In addition, the levels of a number of further inflammatory mediators were increased, when determined by multiplex cytometric bead array in tissue blocks from selected brain areas and in the cerebrospinal fluid (CSF).

Interestingly, the central enrichment in IL-6 showed a strong correlation with its presence in the liver and spleen, whereas levels in the lung were two to three orders of magnitude higher. These latter findings correlated with the prominent respiratory insufficiency observed in the patients contributing to their death. Accordingly, the pattern recognition receptors (PRRs, purinergic P2X7 receptor; toll-like receptor 3, TLR3) and the NLRP3 inflammasome, regulating together the synthesis of IL-1 and - 6, were also up-regulated in the brain and peripheral organs.^5^

In addition to the loss of microglial P2Y12Rs, BBB damage was associated with marked morphological alterations of microglia, especially in the medulla oblongata, apparently also contributing to the imminent neurological malfunction. In the medulla there are a number of autonomic nuclei regulating key bodily functions for example in the respiratory and cardiovascular systems.

Then, single nuclei mRNA sequencing (snRNA-seq) from medullary samples confirmed in the COVID cases the reduced expression of P2Y12Rs and CX3CR1 (fractalkine). The CX3CL1-CX3CR1 axis is functioning via the release of the chemokine CX3CL1 from neurons and its subsequent binding to the microglial receptor, CX3CR1. This chemotactic network functioned perfectly well in control samples, but was completely absent in COVID samples.

Microglial dysfunction resulted in metabolic failure and mitochondrial damage. Especially, different protein complexes involved in the mitochondrial electron transport chain participated in mitochondrial damage. Stimulated emission depletion (STED) microscopic imaging confirmed the intra-mitochondrial localization of cytochrome c (Cytc), in control samples, but in the COVID cases, the efflux of Cytc became evident by registering its reduction in mitochondria, as a signal of apoptosis. Immuno-electronmicroscopy revealed the disruption of the mitochondrial inner and outer membranes of mitochondria, and damage to the cristae (infoldings of the mitochondrial inner membrane).

Further, it was shown that microglial dysfunction accompanies synapse loss and myelin injury in the brain of COVID cases. An increased number of phagolysosomes (fusion of phagosome and lysosome membranes) at sites of severe neuropathologies appeared with engulfment of virus particles and glutamatergic synapses. Post- embedding immunohistochemistry of myelin basic protein identified the loss of myelin compactness, as well as its dilation and curling.

As a next step, proteomics analysis of medulla oblongata tissue homogenates indicated altered expression of many proteins, which are known to regulate core microglial signatures. Single nuclei RNA-seq data revealed changes in microglial activation, oligodendrocyte differentiation and migration, synapse organization, and suggested diverse metabolic changes. Evidence for the involvement of platelet- derived growth factor and vascular endothelial growth factor came from the protein complex analysis, while machine learning identified eight different proteins, also containing the previously recognized pro-inflammatory mediators and PRRs.

The reported findings are of extreme importance, because they give for the first time a rather complete picture of the pathological changes developing in the human brain of COVID cases by the use of standard morphological (light and confocal microscopy) and most contemporary immuno-histochemical methods (immuno- transmission electronmicroscopy, STED microscopy) in combination with correlation analyses and machine learning. A range of standard (quantitative PCR, cytometric bead assay) and more advanced (single nuclei RNAseq, proteomics) approaches were also utilized. This allowed to find out that the complex neuropathologies of the COVID-induced damage are grouped around the microglial alterations, which are the primary causes of the severity and fatal outcome of this disease (Fig. 1).

## References

[1] Fekete, R. et al. Microglia dysfunction, neurovascular inflammation and focal neuropathologies are linked to IL-1- and IL-6-related systemic inflammation in COVID-19. *Nat. Neurosci.* **28**, 558–576 (2025). 40050441 10.1038/s41593-025-01871-z PMC11893456

[2] Wolf, S. A., Boddeke, H. W. G. M. & Kettenmann, H. Microglia in physiology and disease. *Ann. Rev. Physiol.* **79**, 619–643 (2017). 27959620 10.1146/annurev-physiol-022516-034406

[3] Fan, Y., Xie, L. & Chung, C. Y. Signaling pathways controlling microglia chemotaxis. *Mol. Cell* **40**, 163–168 (2017). 10.14348/molcells.2017.0011 PMC5386953 28301917

[4] Allan, S. M., Tyrrell, P. J. & Rothwell, N. J. Interleukin-1 and neuronal injury. *Nat. Rev. Immun.* **5**, 629–640 (2005). 10.1038/nri1664 16034365

[5] Facci, L. et al. Toll-like receptors 2, -3 and -4 prime microglia but not astrocytes across central nervous system regions for ATP-dependent interleukin-1β release. *Sci. Rep.* **4**, 6824 (2014). 25351234 10.1038/srep06824 PMC5381369

